# RACIAL/ETHNIC DIFFERENCES IN SUBJECTIVE COGNITIVE DECLINE AMONG US ADULTS AGED ≥45 YEARS, 2015–2020

**DOI:** 10.1093/geroni/igac059.915

**Published:** 2022-12-20

**Authors:** Benjamin Olivari, Karen Wooten, Eva Jackson, Lisa McGuire, Janet Croft

**Affiliations:** CDC, Atlanta, Georgia, United States; CDC, Atlanta, Georgia, United States; Alzheimer's Association, Chicago, Illinois, United States; CDC, Atlanta, Georgia, United States; CDC, Atlanta, Georgia, United States

## Abstract

Recent studies report that older minorities have a higher prevalence of subjective cognitive decline (SCD), the self-reported experience of worsening or more frequent memory loss or confusion, than older non-Hispanic Whites. This study describes the prevalence of SCD by race/ethnicity and select demographic and social characteristics among U.S. adults aged ≥45 years and whether those reporting symptoms of SCD talked with a health care professional about them. Data collected from 215,406 respondents by the Behavioral Risk Factor Surveillance System (BRFSS) in 2015-2020 were used in the analyses. Statistical comparisons were made using chi-square tests and p value of < 0.05. SCD was reported by nearly 10% of the study population. Asian/Pacific Islanders (5.0%) were least likely to report experiences of SCD and American Indian/Alaska Natives (16.7%) were most likely to report the experience compared to other racial/ethnic groups. Among adults with SCD, Asian/Pacific Islander (34.5%) and Hispanic (40.5%) adults were less likely to talk with a health care professional about their SCD symptoms compared to non-Hispanic White (48.5), non-Hispanic Black (49.5%), and American Indian/Alaska Native (50.5%) adults. Early detection of SCD symptoms can be important to identify early signs of dementia or other potentially treatable conditions and establish a care plan to help people remain as healthy and independent for as long as possible. Health care professionals, especially those working with groups with increased prevalence of SCD, could consider initiating discussions with adults as young as 45 years of age to identify early signs of dementia-like symptoms.

